# Virtopsy of a gravid *Boa constrictor* using computed tomography and magnetic resonance imaging

**DOI:** 10.1016/j.vas.2020.100150

**Published:** 2020-10-08

**Authors:** Dominic Gascho, Udo Hetzel, Nicole Schmid, Rosa M Martinez, Michael J Thali, Henning Richter

**Affiliations:** 1 Institute of Forensic Medicine and Imaging, University of Zurich, Switzerland; 2 Institute of Veterinary Pathology, Vetsuisse Faculty, University of Zurich, Switzerland; 3 Clinic for Zoo Animals, Exotic Pets and Wildlife, Vetsuisse Faculty, University of Zurich, Switzerland; 4 Diagnostic Imaging Research Unit (DIRU), Clinic for Diagnostic Imaging, Vetsuisse Faculty, University of Zurich, Switzerland

**Keywords:** Computed tomography, Magnetic resonance imaging, Boa constrictor, Virtopsy, Postmortem imaging, Virtual necropsy, BIBD, boid inclusion body disease, RAVs, reptarenaviruses, CT, computed tomography, MRI, Magnetic resonance imaging, kVp, kilovoltage peak, mAs, milliampere seconds, TR, repitition time, TE, echo time, ms, milliseconds, IHC, immunohistochemical

## Abstract

This article presents radiologic examinations of a deeply sedated *Boa constrictor* with boid inclusion body disease (BIBD) as an adjunction to the subsequent necropsy. This method is known as virtopsy. The *Boa constrictor* in the present case was gravid. Computed tomography (CT) allowed for the detailed depiction of a fetal skeleton at the rear end of the adult snake. Furthermore, tiny gas formation was detected inside the cranium of the fetus, which was deemed a radiologic sign for decomposition. Magnetic resonance imaging (MRI) delineated the soft tissue at high resolution. This article illustrates the use of CT and MRI for the examination of a gravid *Boa constrictor* before necropsy and demonstrates the detection of “normal” postmortem findings leading to the confirmation of fetal death in situ.

## Highlights

• CT and MRI of a gravid boid inclusion body disease positive Boa constrictor
• CT delineated the fetal skeleton inside the adult snake's body
• Gas formations in the fetal cranium were deemed radiologic signs for decomposition
• MRI delineated the soft tissue and organs of the *Boa constrictor* and its fetus

## GRAPHICAL ABSTRACT

**Figure:**
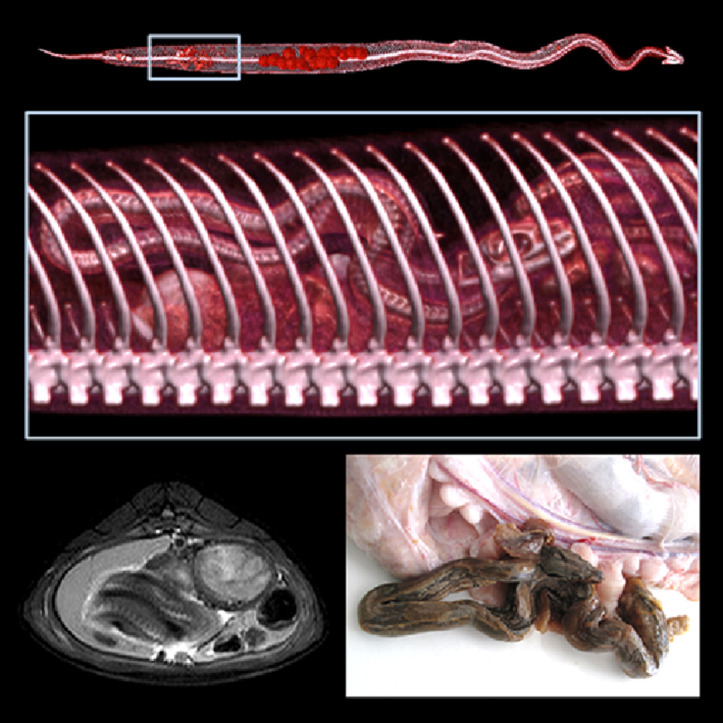

## 1 Introduction

This article describes the radiologic examinations of an infected *Boa constrictor* that was scheduled for a necropsy. The adult *Boa constrictor* was positive for boid inclusion body disease (BIBD), i.e., infected with reptarenaviruses (RAVs), due to demonstration of the characteristic intracytoplasmic inclusion bodies in red and white blood cells from the performed blood smears. There is a strong assumption that RAVs, occurring worldwide in constrictor snakes, cause BIBD, (Stenglein et al., 2017) which is a progressive disease of constrictor snakes and is characterized by cytoplasmic inclusion bodies in a wide range of cell types. BIBD is an often fatal disease (Hetzel et al., 2013). Due to the poor prognosis of the BIBD-positive status, euthanasia and postmortem examinations were scheduled. Since it was assumed that the infected *Boa constrictor* was gravid, additional examinations were conducted to reveal possible lesions and to noninvasively determine the state of gravidity. In contrast to most common snakes, which are oviparous, *Boa constrictors* are viviparous with a true placenta. The embryo of a *Boa constrictor* develops within the salpinx (uterus). After five to eight months of pregnancy, a female *Boa constrictor* gives birth to live offspring (Howard, 2011). In the present case, computed tomography (CT) and magnetic resonance imaging (MRI) of the deeply sedated *Boa constrictor* were performed shortly before the necropsy was conducted. Applying CT and MRI as an adjunction to necropsy is known as a virtual necropsy (or autopsy in the case of humans) or virtopsy (Dirnhofer et al., 2006; Ibrahim, Bakar, & Noordin, 2012). To the best of our knowledge, the use of CT or MRI for the examination of a gravid snake has not been presented in the literature.

This article describes the CT and MRI examination of a deeply sedated, gravid *Boa constrictor* and the in situ examination of a fetus.

## 2 Material and methods

The *Boa constrictor* (weight: 4.3 kg, length: 2 m) was deeply sedated by the administration of ketamine (<25 mg/kg) and medetomidine (<0.3 mg/kg).

A CT scan of the entire body was performed using a 128-slice CT (SOMATOM Definition Flash, Siemens Healthcare, Forchheim, Germany). The scan was performed with 120 kVp using dose modulation (Gascho, Thali, et al., 2018) with an average tube current of 571 mAs. The scan duration was approximately 1 minute. The raw data were reconstructed with a slice thickness of 0.6 mm and increments of 0.4 mm using hard (B60) and soft (B30) kernels.

Following the CT examination, an in situ MRI examination of the fetus was performed using a 3 Tesla MRI scanner (Achieva 3.0 TX, Philips Medical System, Best, The Netherlands). An 8-channel small extremity coil was used for the examination. The examination included T2-weighted sequences in transversal (TR: 5363 ms; TE: 100) and coronal (TR: 4171 ms; TE: 100 ms) orientations. The slice thicknesses were 2 mm. The MRI examination took approximately 15 minutes.

Directly after the radiological examination, the animal was euthanized by overdosing with the sedation mentioned above. A full postmortem examination was performed, and this included histological and immunohistochemical (IHC) investigations of the brain as well as the respiratory, gastrointestinal and urogenital tracts on formalin-fixed paraffin-embedded material (Hetzel et al., 2013).

## 3 Results

**Fig. 1:**
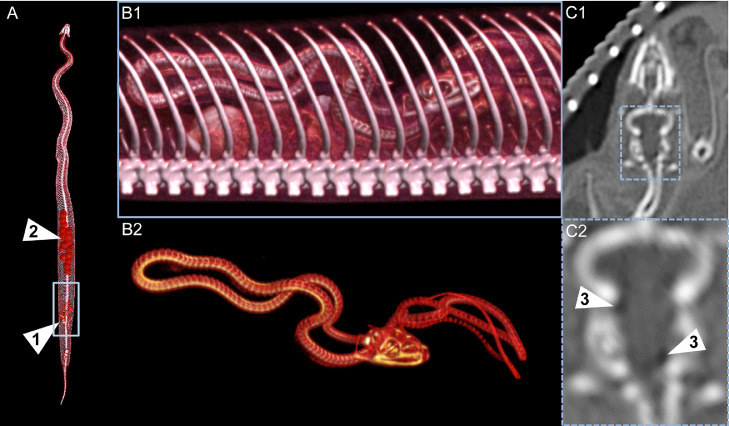
Volume renderings of the entire body of the adult snake (A) and of the fetus in situ (B1-2) as well as the multiplanar reformation aligned to the head of the fetus (C1-2). The fetus was detected in the rear end of the adult snake (arrowhead 1). The volume rendering also highlights the follicles (arrowhead 2). Small gas bubbles were detected in the head of the fetus (arrowheads 3).

**Fig. 2:**
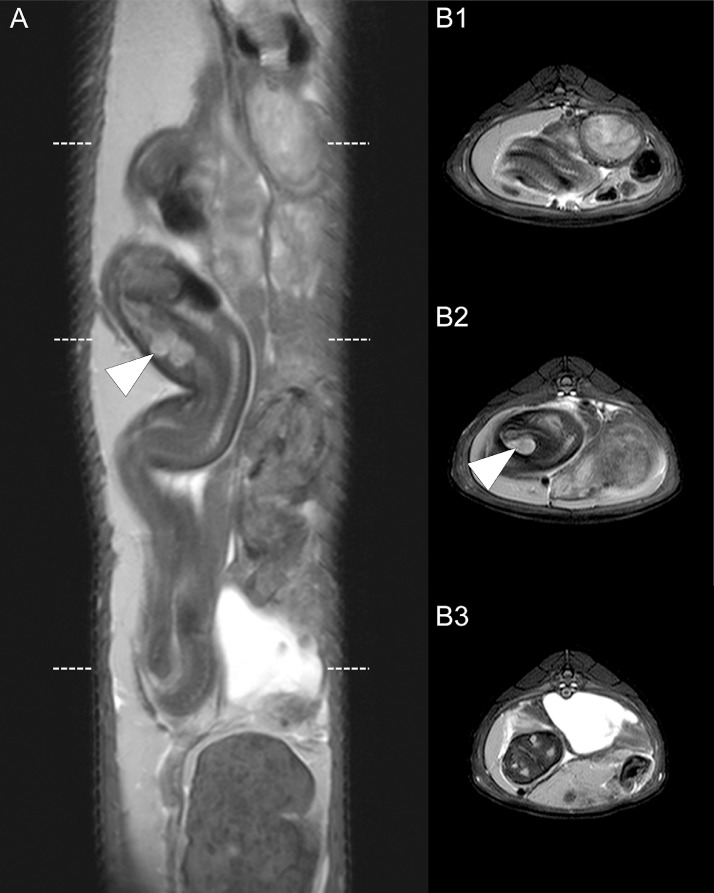
T2-weighted images of the fetus in situ in the coronal (A) and transversal (B1-3) views. The MRI examination displays the soft tissue in high resolution. The arrowhead points to the head of the fetus. The MRI examination did not detect pathologies or injuries.

The CT scan of the adult *Boa constrictor* located a single fetus at the rear end of the adult snake (Fig. 1A). Further postprocessing techniques, including volume rendering, allowed a detailed visualization of the fetal skeleton inside the adult snake's body (Fig. 1B). After a thorough diagnostic assessment using multiplanar reformations, tiny gas formations were detected in the fetal cranium on the CT scan images (Fig. 1C), and these were deemed radiologic signs for decomposition. In addition to the bony structures and gas formations on the CT scan images, the MRI examination delineated the soft tissue and organs of the *Boa constrictor* and its fetus (Fig. 2A and B).

Although gas formations were the only signs of death, their detection inside the cranium was a strong indication that the fetus was already decomposing. Furthermore, it was observed that the fetus did not change its position over the course of the radiologic examinations. Despite the high-resolution CT and MRI, further pathologies or injuries of the adult snake or its fetus were not detected.

The necropsy confirmed that the fetus was dead and slightly decomposed. Further pathologies or injuries were not revealed during necropsy.

Postmortem examination of the adult female demonstrated mild emaciation and hepatic lipidosis. Histological and IHC investigations showed characteristic intracytoplasmic eosinophilic and IHC-positive reactions, respectively. These reactions were predominantly detected in the brain, liver, pancreas, uterus and kidneys. In addition, pyogranulomatous splenitis due to mycobacterial infection was detected. Due to the progressive stage of decomposition, no further investigations of the fetal tissues were performed.

## 4 Discussion

This article illustrates the use of CT and MRI for the examination of a gravid *Boa constrictor* before necropsy and demonstrates the detection of “normal” postmortem findings leading to the confirmation of fetal death in situ.

The constant further developments in the field of radiologic imaging along with an increase in image quality has led to gaining importance of diagnostic imaging of captive snakes for the diagnosis of diseases and injuries (Banzato et al., 2013). The use of radiography and ultrasound is mainly described in the literature, while the use of CT and MRI is much less frequently mentioned (Banzato et al., 2013). The use of CT scanning was presented for the diagnosis of fractures (Rahal et al., 2011) or the investigation of the lung (Pees et al., 2007). MRI, in turn, was applied for neurologic examinations (Mariani, 2007) or investigations of the gastrointestinal tract and other visceral organs (Hansen et al., 2013). In addition to the diagnosis of diseases and injuries in (clinical) veterinary radiology (Dennis, 2003; Labruyère & Schwarz, 2013) or the noninvasive visualization of the anatomy for biological purposes (Lauridsen et al., 2011), CT and MRI are valuable imaging modalities for noninvasive adjunction or alternative examination methods for necropsy (Thali et al., 2007; Munro & Munro, 2011; Lee et al., 2011; Franckenberg et al., 2015; Watson & Heng, 2017; Gascho, Bolliger, et al., 2018). The virtopsy approach describes a new and rapidly evolving noninvasive examination procedure involving the use of modern imaging modalities (Dirnhofer et al., 2006). This approach is based on the idea that radiologic examinations before a body orifice provide additional information on the case. In the present case, CT presented decomposition-related gas formations in accordance with the mummified state of the fetus. Further radiologic or macroscopic findings were not detected. BIBD is thought to be immunosuppressive, and pathological alterations, e.g., pneumonia or enteritis, often occur only in late stages of the disease. Thus, the absence of radiologic and macroscopic lesions is not unusual. Hence, vertical, i.e., transplacental transmission of reptarenaviruses is known in *Boa constrictors* (Keller et al., 2017); thus, fetal death could be related to maternal BIBD and viral infection.

**Fig. 3:**
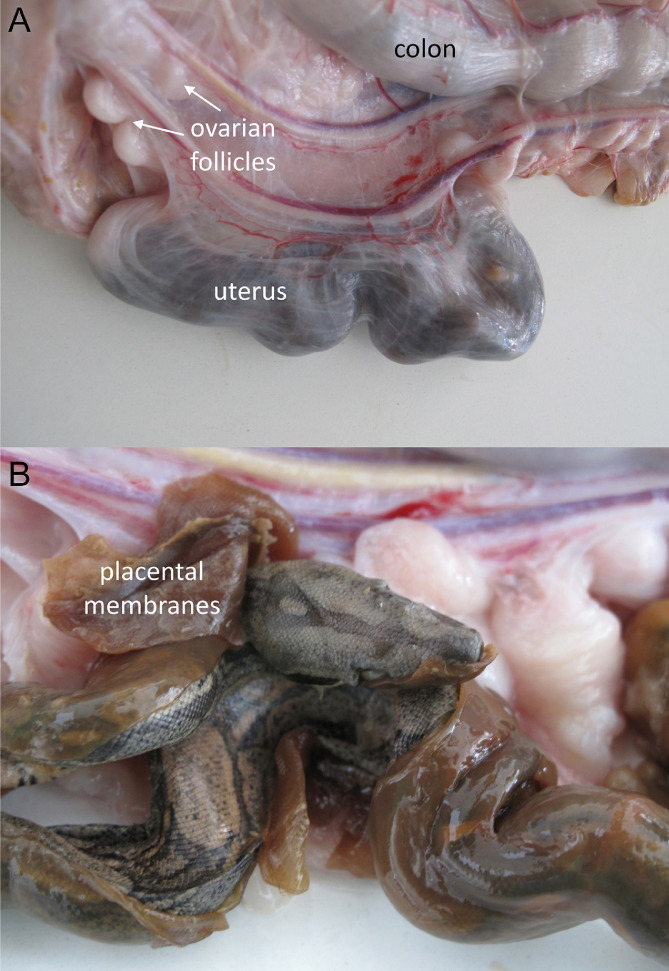
Fetus in the intrauterine location (A). The gravid uterus of a *Boa constrictor* is thin walled, comparable to a 1-2 mm thin membrane. The dead fetus (B) was partly covered by mucinous degenerated placental membranes.

In summary, this article presents high-resolution imaging of a *Boa constrictor* fetus in situ by CT and MRI. These radiologic modalities allow for not only imaging the anatomy, pathologies and injuries but also detecting “normal” postmortem findings, such as decomposition-related gas, which allowed for determining the death of the fetus in the present case. Fig. 3.

## Conflict of Interest Statement

None of the authors has a real or perceived conflict of interest in any of the material that is presented in the manuscript. The authors have no conflicts of interest to report. This scientific paper received no external funding.

## Ethical Statement

Ethical approval is not required for this case report. No animals were killed for the scientific purpose of this study.
